# Gemcitabine plus oxaliplatin for the treatment of leptomeningeal metastases of non-small cell lung cancer: A case report and review of the literature

**DOI:** 10.3892/ol.2013.1263

**Published:** 2013-03-19

**Authors:** CHONG BAI, HUI SHI, DAN LIU, TIANYI ZHU, ZHENLI HU, QIANG LI

**Affiliations:** Department of Respiratory Medicine, Changhai Hospital, Second Military Medical University, Shanghai 200433, P.R. China

**Keywords:** non-small cell lung cancer, leptomeningeal metastases, gemcitabine, oxaliplatin

## Abstract

A 62-year-old male presented with stage IV lung adenocarcinoma with leptomeningeal metastases (LM). Gemcitabine (1000 mg/m^2^ i.v.) was administered on days 1 and 8 while oxaliplatin (100/m^2^ i.v.) was administered on day 1 and repeated for 4 cycles every 3 weeks. Computerized tomography (CT) and cerebrospinal fluid (CSF) were used to evaluate the response of the LM and the primary tumor to drug therapy. Following the administration of chemotherapy, headaches were observed to be notably reduced 6 days later and absent after 14 days. The symptoms of coughing and chest pain were alleviated. Subsequent to 4 cycles of treatment, the patient had a partial response (PR) and the CSF pressure was normal. Analysis of the CSF revealed that it was colorless, positive for protein, had a total cell number of 0/l and contained no cancer cells. However, the primary lung tumor progressed for 1 year. This may suggest that first-line therapies, including the use of gemcitabine and oxalipaltin, may be appropriate for the treatment of non-small cell lung carcinoma (NSCLC) with LM involvement.

## Introduction

Leptomeningeal metastasis (LM) occurs when cancer cells spread to the meninges, the layers of tissue that cover the brain and spinal cord. Metastases spread to the meninges through the blood or carried by the cerebrospinal fluid (CSF) that flows through the meninges (1). The incidence rate of LM is ∼5% worldwide, with a poor prognosis. The median survival of patients with LM is ∼3 months (2,3) and the current treatment methods include localized radiation therapy, intrathecal chemotherapy or systemic chemotherapy (1). Non-small cell lung carcinoma (NSCLC) consists of any type of epithelial lung cancer other than small cell lung carcinoma (SCLC). The present case report describes a patient with LM from SCLC who responded to gemcitabine plus oxaliplatin. The procedure followed complied with the ethical standards of the Changhai Hospital Institutional Review Board (IRB) and was approved by the hospital committee. Informed written consent was obtained from the subject.

## Case report

**Figure 1:**
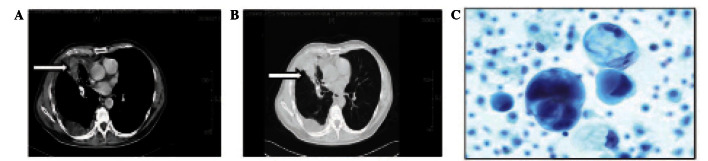
Non-small cell lung cancer. (A and B) Transverse contrast-enhanced CT scan obtained with (A) lung and (B) mediastinal window, at the level of distal tracha, revealed a mass (2cm×1.5cm, arrow) in the right upper lobe and some lymph nodes, a small sized right effusion. Adenocarcinoma cells (C) were found in the smears of the pleural effusion (H&E staining; magnification, ×40).

**Figure 2:**
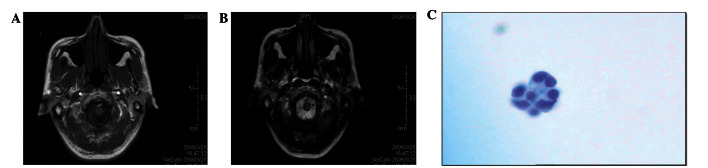
Leptomeningeal metastasis. (A) T2-weighted image revealing widening of the vermian sulci. (B) Post-contrast T1-weighted image revealing enhancement of the vermian sulci corresponding to leptomeningeal infiltration. (C) Adenocarcinoma cells were observed in the cerebrospinal fluid (CSF). (H&E staining; magnification, ×40)

**Figure 3:**
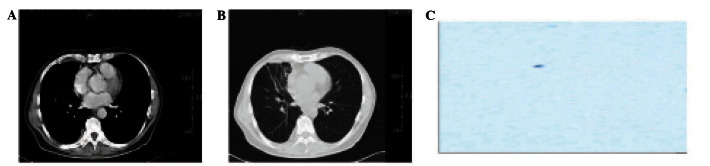
Following chemotherapy. (A) and (B) Computerized tomography (CT) showing the 0.5×0.5 cm shadow in the right lower lung and the thickening of the pleura of the right side of the chest. (C) No cancer cells were detected in the cerebrospinal fluid (CSF).(H&E staining; magnification, ×40)

A 62-year-old male patient (weight, 65 kg; height, 166 cm) was admitted to Changhai hospital, The Second Military Medical University (Shanghai, China), due to coughing and chest pain that had occurred for 5 months. The patient had suffered an unexplained dry cough since September 2005, accompanied by chest tightness and pain. In March 2006, a chest X-ray showed a shadow in the right lower lung with a small amount of pleural effusion. The chest computerized tomography (CT) showed a 2×1.5 cm block shadow in the right lower lung, a medium dose pleural effusion in the right chest cavity and certain mediastinal lymph nodes with calcification (Fig. 1A and B). The emission CT (ECT) showed numerous bone metastases. On March 27th, 2006 (week 0), a tube was placed in the right chest cavity and drained 2400 ml of the pleural effusion. The entire pleural effusion was drained after 3 days and consisted of ∼3,020 ml in total. Adenocarcinoma cells were identified in smears of the pleural effusion (Fig. 1C) and the diagnosis from a Board Certified Pathologist was determined as that of a right lower lung adenocarcinoma (T4N2M1, stage IV). Following admission, the patient began to develop a severe headache with nausea and vomiting but without cranial and spinal nerve dysfunction, or signs of leptomeningeal irritation, such as Brudzinski’s or Kernig’s sign. There were no abnormal signs in the head magnetic resonance (MR; Fig. 2A and B) or gastroscopy images. In the first week, a lumbar puncture was performed and the pressure of the CSF was 18 cm H_2_O. The result of the test was colorless, positive for protein, had a total cell number of 10×10^6^/l and contained cancer cells (Fig. 2C) (4). Chemotherapy was started with 1.8 g/day gemcitabine (from days 1–8) and 200 mg oxaliplatin (on day 1 only). The headache symptoms were notably eased after the first week and disappeared completely in the second week. The symptoms of coughing and chest pain were also alleviated. Chemotherapy was administered again in weeks 4 (cycle 2), 7 (cycle 3) and 9 (cycle 4). In week 12, the pressure of the CSF was 12 cm H_2_O. The CSF analysis was colorless, positive for protein, had a total cell number of 0/l and contained no cancer cells (Fig. 3C). The CT showed that the shadow in the right lower lung was 0.5×0.5 cm and that the pleura of the right chest was thickened (Fig. 3A and B). Another 4 cycles of chemotherapy were administered. The patient was monitored by monthly visits until January 30th, 2007 (week 44). During this time the patient was stable. On April 29th, 2007 (week 57), the primary tumor in the lung was observed to have progressed and 250 mg gefitinib a day was administered.

## Discussion

A number of malignancies may cause LM, among which breast, lung [particularly adenocarcinoma (5)] and urinary tract tumors are the most common and account for ∼80% (6). An undifferentiated pathology type and other distant metastases are independent risk factors for LM (7). The clinical features of LM may include an intractable headache, nausea, vomiting, dizziness and alterations in mood and consciousness (8). In cases where the cranial nerve is attacked, symptoms may include diplopia, impaired vision, facial numbness, difficulties in swallowing and abnormalities in tasting and hearing (9). In addition, there may also be disruption to the cauda equina, pseudomeningitis or no symptoms at all (10). The most common symptoms are headaches, mental and behavioral changes (often first detected by family members) and facets of higher cortical functions, including impaired comprehension, reading, calculation and difficulty performing motor function tasks, such as eating or dressing. Acute or rarer symptoms, including seizures (10–15%) or intratumoral bleeding (10%), are more common in metastatic melanomas (11). The gold standard for the identification of LM is identifying tumor cells of the same pathological type as the primary focus. The main symptoms of the patient in the present case were a stubborn headache, nausea and vomiting. Tumor cells were observed in the CSF sample. Therefore, the diagnosis of this patient was confirmed.

For patients with LM, few therapeutic options are available other than palliative measures, which include whole brain irradiation or intrathecal chemotherapy (12). LM from NSCLC is a difficult disease to treat and remains a major obstacle in the clinical course of NSCLC. Although the majority of the clinical chemotherapy drugs are not able to get through the blood-brain barrier, the present study observed that systemic anti-cancer drugs prolonged the survival period and rates of patients with LM from NSCLC. This may be as the transfer of LM disrupts the blood-brain barrier, which allows the chemotherapy drugs to penetrate into the brain and function there. Drugs such as methotrexate, cytarabine and thiotepa are not the most efficient for NSCLC patients, so they are no longer used for the treatment of NSCLC with meningeal metastasis. At present, patients receiving first-time treatment often receive platinum-based drugs in combination with gemcitabine, paclitaxel, vinorelbine or other treatments, which have achieved a higher remission rate (13).

Few cases report that these first-line chemotherapies have been used for the treatment of NSCLC patients with LM (14–16). In the present study, the stage IV NSCLC patient, who received gemcitabine plus oxaliplatin for four courses, achieved a partial remission and the LM was controlled.

This study suggests that first-line chemotherapy using gemcitabine and platinum-containing drugs may be effective for the treatment of NSCLC patients with LM. In addition, the epidermal growth factor receptor (EGFR) and tyrosine kinase inhibitors (TKI), including gefitinib and erlotinib, have been shown to have greater efficacy in LM (17). These new molecularly targeted drugs may act as another treatment option. However, further research is required (18–20).
